# INPP5D inhibits anti-malarial immunity by promoting IRF3 degradation through selective autophagy

**DOI:** 10.1080/27694127.2023.2254614

**Published:** 2023-09-18

**Authors:** Hongyu Li, Xiao Yu

**Affiliations:** Department of Immunology, Guangdong Provincial Key Lab of Single Cell Technology and Application, School of Basic Medical Sciences, Southern Medical University, Guangzhou, China

**Keywords:** Anti-malarial immunity, INPP5D, IRF3, selective autophagy, type I interferon

## Abstract

As a member of the inositol polyphosphate-5-phosphatase family, INPP5D (inositol polyphosphate-5-phosphatase D) is an important regulator of immune cell activation. To date, the mechanisms underlying anti-malarial immunity have not been elucidated. We recently identified INPP5D as a negative regulator of IFN-I (type I interferon) signaling by promoting autophagic degradation of IRF3 (interferon regulatory factor 3) during malaria infection. Mechanistically, INPP5D enhances the association between IRF3 and the autophagy receptor CALCOCO2/NDP52 (calcium binding and coiled-coil domain 2), which promotes the K63-linked ubiquitination of IRF3 at K313 and serves as a signal for CALCOCO2-dependent selective macroautophagy (hereafter autophagy). Moreover, INPP5D is downregulated by IFN-I-induced miR-155-5p after *Plasmodium yoelii* (*P. yoelii*) *nigeriensis* N67 infection and plays a role as a feedback loop between IFN-I signaling and autophagy. Thus, our study reveals the key role of INPP5D in mediating the crosstalk between IFN-I response and autophagy during anti-malarial immune responses, and suggests that INPP5D may be a potential therapeutic target to control malaria.

**Abbreviations**: ATG5; autophagy-related 5; CALCOCO2/NDP52, calcium binding and coiled-coil domain 2; CQ, chloroquine; INPP5D/SHIP1, inositol polyphosphate-5-phosphatase D; IRF3, interferon regulatory factor 3; IFN-I, type I interferon; 3-MA, 3-methyladenine.

Malaria is caused by *Plasmodium* infection and remains a serious disease that affects millions of people worldwide. The human parasites *P. falciparum* or *P. vivax*, and the murine parasites *P. yoelii* or *P. berghei* infection triggers IFN-I signaling, which is tightly controlled and plays a critical role in host innate immunity. To identify targets for effectively curing malaria, it is essential to investigate the control of IFN-I in malaria more precisely. Multiple regulators are involved in the regulation of IFN-I production during malaria. The negative regulators, in particular, are essential not only to maintain the immune homeostasis in the organisms, but also have key roles in the escape mechanisms of pathogens when subverted. Despite ongoing research, the molecular mechanisms which responsible for most of the immune responses remain unclear. Our recent research revealed the downregulation of a host gene *INPP5D* in *P. falciparum*-infected patients. This suggests that INPP5D may have a role in anti-malarial immunity. Furthermore, we observed higher levels of IFN-I and greater resistance to *P. yoelii nigeriensis* N67 infection in a mouse model with genetically ablated *Inpp5d*. Otherwise, this phenomenon disappeared in *Ifnar* (type I interferon receptor) knockout mice. Overall, we suggested that the genetic deletion of *Inpp5d* provides stronger protection during host anti-malarial immune response, which depends on IFN-I-IFNAR signaling.

The protein INPP5D performs multiple functions through distinct mechanisms. However, the specific role of INPP5D in anti-malarial immunity is unclear. In our study, we uncovered that INPP5D functions as a negative regulator in IFN-I signaling and demonstrated that this protein is downregulated by IFN-I-mediated miR-155-5p expression (Figure 1). Phosphorylation of IRF3 is a key step during IFN-I signaling activation. In the unstimulated state, IRF3 is located in the cytoplasm. It becomes phosphorylated at specific serine residues after stimulation or infection, which activates it, allows it to form dimerization, translocate into the nucleus, and initiate IFN-I signaling upon infection. The phosphorylation of IRF3 is significant in determining the immune status against an infection. Our study demonstrated that INPP5D deficiency leads to an increase in phosphorylation of IRF3, resulting in enhanced IFN-I production and improved host anti-malarial immunity after *P. yoelii nigeriensis* N67 infection.

**Figure 1.**
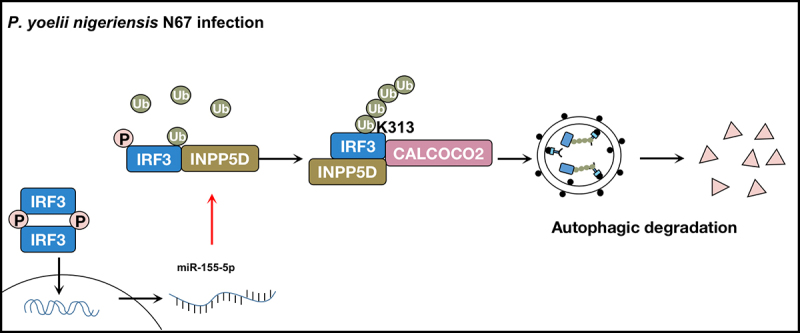
A proposed working model to illustrate the negative feedback loop of IFN-I signaling generated by IRF3-miR-155-INPP5D-CALCOCO2 axis in anti-malarial immunity. During *P. yoelii nigeriensis* N67 infection, INPP5D interacts with phosphorylated IRF3 and enhances K63-linked poly-ubiquitination of IRF3 at K313, thereby stabilizing the interaction between IRF3 and its selective autophagy receptor CALCOCO2. Binding between INPP5D, IRF3, and CALCOCO2 strengthens the autophagic degradation of activated IRF3, depleting IFN-I responses against *P. yoelii* infection. Additionally, IRF3-dependent IFN-I response activated by *P. yoelii* gDNA and RNA induces miR-155-5p expression to downregulate INPP5D, acting as a negative feedback loop between IFN-I signaling and autophagy.

After observing that INPP5D inhibits the phosphorylation of IRF3, we conducted further investigations and discovered that INPP5D accelerated the degradation of IRF3. The proteasome and the lysosome/autophagy are the two main systems that are used to control protein degradation. To further investigate the degradation mechanism of IRF3 induced by INPP5D, we used different inhibitors, including the autophagy inhibitor 3-methyladenine (3-MA), the non-specific autophagy inhibitor chloroquine (CQ), and the proteasome inhibitor MG132. We observed that 3-MA and CQ, but not MG132, blocked the turnover of IRF3 triggered by INPP5D. Importantly, the degradation of IRF3 induced by INPP5D was also abolished in *atg5^−/−^* and *beclin1^−/−^* cells. These results suggested that INPP5D promotes the autophagic degradation of IRF3. We also found that INPP5D can interact with phosphorylated IRF3 and since INPP5D is not an autophagosomal cargo, we decided to identify the potential selective autophagy receptor responsible for the autophagic degradation of IRF3. We established that INPP5D can promote the interaction between IRF3 and the selective autophagy receptor CALCOCO2. Furthermore, we discovered that INPP5D binds to phosphorylated IRF3 and promotes K63-linked poly-ubiquitination on IRF3 at K313, which strengthens the binding between IRF3 and CALCOCO2, enhancing the targeting of activated IRF3 to autophagic degradation (Figure 1).

Autophagy is a degradative pathway that is conserved across eukaryotic species. The autophagy inhibitor CQ has many uses, including having anti-malarial, anti-viral, anti-bacterial, anti-protozoan, anti-autoimmunity, and anti-cancer effects. However, the mechanism by which CQ operates in different diseases is not well understood since in not exclusively inhibiting autophagy. CQ’s involvement in diseases motivates us to investigate the possible crosstalk between autophagy and IFN-I signaling. Autophagy is a double-edged sword in infectious disease, it can lead to the production or degradation of critical IFN-I signaling molecules. Therefore, understanding the interaction between autophagy and IFN-I signaling in infectious diseases is crucial. Our current study demonstrated that INPP5D strengthens the association between CALCOCO2 and IRF3, and promotes the autophagic degradation of activated IRF3, leading to a decrease in the production of IFN-I and inhibiting immune responses against *P. yoelii nigeriensis* N67 infection.

In conclusion, the highlight of our work is the discovery of a novel role for INPP5D in regulating anti-malarial immunity negatively. In particular, our study shows the significance of INPP5D in mediating the crosstalk between IFN-I response and autophagy in anti-malarial immune responses. Although this mechanism lacks sufficient clinical data, our work suggests that targeting autophagy could be an effective strategy to modulate anti-malarial immunity ^1^.
